# Concerning Woman Suffrage

**Published:** 1917-07-07

**Authors:** 


### CONCERNING WOMAN SUFFRAGE.

By CADUCEUS.

The well-earned growth in influence of the medical profession is nowadays acknowledged. Ecclesiastics own that they are being given the go-by. Of the religion of humanity the doctor is almost the high-priest, for he has shown how to exorcise some of the devils of disease which possess it. The clinician pushes therapeutics further and further, while the investigator lays down plans for the rank and file of preventive medicine to follow in making the people healthy. But the field open to students of medicine stretches far beyond all this. So many departments of human activity are concerned, whether the fact be realised or no, with physiology, with special psychology, with abnormal psychology, with heredity normal or pathological, all subjects lumped in with medicine, that it seems likely that the medical profession a century hence may have as its province a far larger proportion of reasoned human conduct than it has at present. And a good thing too, if medicine but takes up the task with energy and disinterestedness, is not spoilt by the favour of the rich and the great and the powerful, remembers and resumes its old unconforming character. For many of the problems encountered on the borderland of medicine will be of a delicate, controversial nature, and the direction in which their solution is sought may seem wrong. One may have—strange fate for the medical spirit! —to be reactionary. As, for instance, in our present subject of woman suffrage. The general urge towards democracy, prompted by the pass in which Europe finds itself under oligarchy, naturally favours political power for the weaker vessel, and the more numerous vessel, of womankind. But the intrinsic merits of that particular cause may not do so.

Of course, the opinions expressed here commit The Hospital to nothing. Editors nowadays fall for the most part into two classes. The first thinks of the circulation of his journal; of quantity merely. But the second, of its quality in an exacting sense of that word, of its real and abiding worth. And as the aims of the two schools are opposed, so also are their methods, especially in one particular. To get notoriety, the more one-sided and extreme, the more "yellow" a print is, the better. But to get respect some impartiality must be shown. The two-faced Janus was a deity of higher rank even than Jove, and his aid was invoked before that of the thunderbolts. Now (talking of Olympian personages) the editor of this present publication has always seemed to belong to the latter of the two schools indicated above. One has often seen in these columns a question or a theory attacked one week by one contributor and alluded to rather approvingly the next by another. Somewhere in the mean of these oscillations truth is obviously more likely to be found than in either extreme, just as a ship's true course is kept by alternate movements of the helm to port and starboard, whereas lashing the wheel hard over means merely moving in a circle. Let those who burn argue with those who adore (although, of course, we all adore womankind), and then, most times, we shall see which antagonist is nearer the right—a result surely worth the little friction necessarily involved.

And so let us not be afraid of questioning decisions, even in this matter of woman suffrage, which, besides the general medical interest already claimed for it, has also a particular medical interest. Three lady doctors out of four are suffragists, it has been said; and up to the present this writer has found the odd one of considerably higher intelligence than the other three. More important than that, municipal medical work is growing—or was growing—every year, and women seek seats on municipal committees with eagerness; if, then, electoral equality with men be granted them, their standing on such committees will be proportionately raised. More important again, very much of legislation has to do with medicine, and the feminine vote, which after the slaughter of the war will more than ever outnumber the male, will have to be placated by the devisers of social reform measures. Now, the writer would like, if he may, to say at once that he is no Tory. But all the same he is unwilling to see sciolism, let alone the crude, sentimental, suicidal notions of, say, a Mrs. Carrie Nation, let loose on problems the solution of which demands highly developed study. But, before arguing generally on the subject, one must forestall those who claim that the war has altered the question of woman suffrage out of recognition. The war is a huge thing, no doubt; but there have been wars before in which just as large a proportion of the able-bodied male population has gone fighting, leaving the women behind to practise all manner of arts usually strange to them. What is perhaps unprecedented is the amount of remuneration received by them for doing so. Can anyone see munition girls mafficking on a railway platform, or tram conductresses chaffing their passengers, or working-class girls happily preening themselves in their nurse-probationers' uniforms, or *chauffeuses* getting a male bystander to crank up a cold engine for them, or lady *locum* clerks making hearty lunches and smoking before the fire in cafés, and not perceive that the feminine spirit, which rejoices most of all in two things—gregariousness and (on Chaucer's authority) getting its own way—is not excellently suited by masculine work and pay, which procures both these heart's desires? To give the bulk of women war-workers credit for self-sacrifice is merely the so abundant cant of the newspapers and the political platform, once faithfully characterised thus:

"The better it is the worse it is, the kind is so inferior. It has nothing to do with the truth or the search for it; nothing to do with intelligence, or candour, or honour. It's an appeal to the love of hollow, idiotic words, of shutting the eyes tight and making a noise."

The women who got in the harvest after Flodden was fought were made heroines of by nobody, and very rightly.

This cant it is, too (for years before the war all criticism in this country was more or less soapy), which disguises the real reason for denying women the vote—namely, their comparative want of capacity. In a speech hailed as "great," one of the numerous tribe of lawyers in the House of Commons dwelt on the achievements of Joan of Arc and Mrs. Siddons. As a matter of fact, there were hardly any feminine names of other note for him to mention. The ascertained facts, and it is for medicine to add to their number, as to the intellectual secondary sexual characters are not in much dispute. Practically the chapter written by Mr Havelock Ellis—who as a woman-suffragist will not be accused of adverse prejudice—on the subject may be summed up in Mr. George Moore's notable judgment:—

"Women have succeeded as actresses and as courtesans, and as saints; yes, most of all as saints. They have worshipped worthily the gods that men have created."

But which has stood best the test of time, the passive emotionalism, extreme in character, and plainly based on the physical, of St. Teresa, or the wisdom of St. Augustine? Only in the lowest of the arts—namely, acting, and (I have not seen this noted before) in long-distance swimming—are women in the same class as men. The lady who one hopes will reply to these remarks—she will get the last word—will not quarrel with our ruling out a physical accomplishment; and so we are left with histrionic ability as the one certain claim feminine intelligence has to be ranked equal with the masculine one. There are women of talent, of course, but curious it is to note how many, at any rate nowadays, married or came under the influence of men who had already made a name for the same kind of work. Madame Curie, Mrs. Sidney Webb, Lady Gregory, are well-known instances of this association which occur on the spur of the moment. A similar phenomenon is not noticed in the case of male celebrities. And if it be urged that woman's backwardness is the result of disabilities imposed on her, why, what oppressions could be compared with those through which the fiery soul of male genius has worked its way, victoriously fighting the world and its own broken health, to get its task but done? Of what importance are the efforts of others either to help or hinder a genuine original capacity? Galton's G class of ability is, he says, independent of education. In spite of a modern access of opportunity, the only woman "torch-bearer" (with one possible exception to be mentioned shortly) at present apparent is in a picture merely—namely, Mr. Walter Crane's rendering of the famous Lucretian image. We have had quite two generations of "lady meds." now. Well, Mrs. Scharlieb is a fine operator, and Lydia Rabinowitsch and Miss Lane Claypon are reliable researchers of the Baconian kind. Medicine would not have been poorer, however, had none of these gifted ladies been born; there would have been someone else to do all their work. And as men outrank women in the aristocracy of intellect, so do they in the middle class of the same. Why does the medical woman defer so to one, I mean before she has any opportunity of discovering justification or lack of justification for her attitude? The woman teacher—of quite young children—is in a different category, for she is in her own province of work, for which she is fitted by Nature, not hindered. To Madame Montessori we are inclined to listen with respect, but not to the writers of silly little books on social medicine. Woman, her emotionalism, her self-sacrifice, is explained by the child, and will be for ages yet in spite of the decline in marriage prospects the last few decades have brought. Preserve us, says that clever miniaturist, Mr. Oliver Onions, from the young man who thinks the New Woman any different from the old one.

Our argument draws to its close. The demerit of all electorates is a low standard of intelligence. Why make this worse by admitting women, whose intelligence on an average is below that of men? A few women are more intelligent than the average man, and some are as intelligent: all these should have voting power. But hard cases make bad law, and whereas the dividing line of sex is immutable and ready drawn by Nature, a franchise standard of intelligence would be almost impossible to ascertain, and quite impossible to enforce with any chance of general agreement therewith. Finally, there are some exceptionally clever women (see just recently again Mrs. Humphry Ward) who probably see that a feminine talent gets recognised much more easily than a masculine one, and who certainly want no vote. Last of all (a postscript indeed of the traditional feminine kind), hear Goethe in his master work:—

Swift they go and swift they go,
And gain a thousand steps or so,
But slow is swift and swift is slow.
Woman may hustle and woman may bustle
But in the end she loses the day,
At one long bound man clears the way.

